# Rotating Magnetic Fields Inhibit Mitochondrial Respiration, Promote Oxidative Stress and Produce Loss of Mitochondrial Integrity in Cancer Cells

**DOI:** 10.3389/fonc.2021.768758

**Published:** 2021-11-10

**Authors:** Martyn A. Sharpe, David S. Baskin, Kumar Pichumani, Omkar B. Ijare, Santosh A. Helekar

**Affiliations:** ^1^ Kenneth R. Peak Center for Brain and Pituitary Tumor Treatment and Research, Houston Methodist Hospital, Houston, TX, United States; ^2^ Department of Neurosurgery, Houston Methodist Hospital, Houston, TX, United States; ^3^ Department of Neurosurgery, Houston Methodist Research Institute, Houston, TX, United States; ^4^ Department of Neurosurgery, Weill Cornell Medical College, New York, NY, United States

**Keywords:** radical pair mechanism, cancer, oxygen consumption, electron transport chain, diffuse intrinsic pontine glioma

## Abstract

Electromagnetic fields (EMF) raise intracellular levels of reactive oxygen species (ROS) that can be toxic to cancer cells. Because weak magnetic fields influence spin state pairing in redox-active radical electron pairs, we hypothesize that they disrupt electron flow in the mitochondrial electron transport chain (ETC). We tested this hypothesis by studying the effects of oscillating magnetic fields (sOMF) produced by a new noninvasive device involving permanent magnets spinning with specific frequency and timing patterns. We studied the effects of sOMF on ETC by measuring the consumption of oxygen (O_2_) by isolated rat liver mitochondria, normal human astrocytes, and several patient derived brain tumor cells, and O_2_ generation/consumption by plant cells with an O_2_ electrode. We also investigated glucose metabolism in tumor cells using ^1^H and ^13^C nuclear magnetic resonance and assessed mitochondrial alterations leading to cell death by using fluorescence microscopy with MitoTracker™ and a fluorescent probe for Caspase 3 activation. We show that sOMF of appropriate field strength, frequency, and on/off profiles completely arrest electron transport in isolated, respiring, rat liver mitochondria and patient derived glioblastoma (GBM), meningioma and diffuse intrinsic pontine glioma (DIPG) cells and can induce loss of mitochondrial integrity. These changes correlate with a decrease in mitochondrial carbon flux in cancer cells and with cancer cell death even in the non-dividing phase of the cell cycle. Our findings suggest that rotating magnetic fields could be therapeutically efficacious in brain cancers such as GBM and DIPG through selective disruption of the electron flow in immobile ETC complexes.

## Introduction

Electromagnetic fields (EMF) are known to produce anticancer effects *in vitro* and *in vivo* (1). We have shown recently that spinning oscillating magnetic fields (sOMF) produced by rotating permanent magnets in a noninvasive magnetic stimulation device (Oncomagnetic Device), developed by us, rapidly kill patient derived glioblastoma (GBM) and non-small cell lung cancer cells in culture (2). We have also shown that this is due to rapid increase in the reactive oxygen species (ROS) in the mitochondria to cytotoxic levels only in cancer cells, and not in normal human cortical neurons, astrocytes, and bronchial epithelial cells (2). However, the biophysical mechanisms underlying these effects, and previously observed anticancer effects by others, are not clear. Magnetic fields at low intensities have pronounced effects on unpaired electrons in molecules participating in chemical reactions and electron transfer processes (3, 4). Pairing of electrons in bimolecular reactions and electron transfer processes with free radical intermediates, termed as the radical pair mechanism (RPM), is perturbed by magnetic fields in the milliTesla (mT) and microTesla (µT) ranges (3–6). This perturbation is due to a quantum mechanical phenomenon in which the spin of an electron tends to align itself with the axis of a magnet. Because pairing of electrons in chemical reactions involving radicals generally require them to be of opposite spins, a magnetic field of µT or mT strength can substantially alter both the product yield and the rate of these reactions (3). An electron transfer process can also be similarly affected (7, 8). More specifically, during normal interactions between free radicals, the dynamics of RPM consist of electron pairs interconverting between opposite spin (↑↓) state (the “singlet” configuration) and like spin (↑↑ or ↓↓) state (the “triplet” configuration) (9, 10). The singlet-triplet interconversion rate of an electron is strongly influenced by an externally applied magnetic field and the magnetic fields of its neighboring atomic nuclei (hyperfine interactions).

While RPM and its modulation by magnetic fields has been studied in chemistry since the 1970’s, its role in biological processes has received attention only in recent decades. Work in this area so far has focused on effects on photosynthesis, protein folding, and a few enzymatic reactions in artificially configured settings (11–13). A few exceptions are the more recent discovery of flavin containing proteins called cryptochromes in the retina of migratory birds and their role in seasonal migration, guided by the µT range magnetic field of the Earth, as well as some other instances of magnetoreception in plants and other organisms in which these flavoproteins play a physiological role (14–17). All the observed effects so far have been with a static magnetic field, or an oscillating magnetic field induced in electromagnetic coils with a single fixed orientation. This works well for measuring effects on reaction rates and yields involving moving molecules in solution that can freely orient themselves in all directions. It applies also to molecules in fixed orientations that rely critically on the direction of the magnetic field or require the perception of its direction, as in the case of avian migration. However, it may not be effective for producing maximal magnetic field effects on biochemical reactions or electron transfer processes involving molecules in fixed positions and orientations, such as the redox centers in the mitochondrial respiratory chain complexes.

To account for the ROS-inducing action of sOMF we have proposed the hypothesis that intermittent patterns of magnetic field oscillations with specific timings in the mT range of field strengths and the super-low range of frequencies disrupt electron flow in the mitochondrial electron transport chain (ETC) complexes because of the magnetic perturbation of RPM. Here, we explore the effects of mT range sOMF produced by rapidly spinning permanent magnets on the electron transport occurring in these complexes. We achieve this by using a sOMF producing component called an oncoscillator of a newly developed potentially therapeutic noninvasive anticancer device called an Oncomagnetic device. This component is comprised of a high field strength neodymium permanent magnet attached to the shaft of a battery-operated electric motor. Sweeping through different rotation frequencies by cyclically turning the motor on and off also allows us to investigate the possible effects of resonance of the sOMF with the periodicities of the electron transfer process and hyperfine interactions. We have used the Oncomagnetic device to successfully treat an end-stage glioblastoma patient under an FDA-approved compassionate use protocol (18).

In a recent publication we have shown that oncomagnetic sOMF have a differential effect on cancer cells, when compared with cells derived from normal tissues (2). sOMF stimulation of GBM and lung cancer cells causes death, and cell death is associated with mitochondrial superoxide generation, oxidation of the glutathione, an increasing GSSG/GSH ratio and the activation of the apoptotic caspase-3/7 cascade. We also show that the sensitivity of cells toward sOMF can be modulated by manipulation of its pro/anti-oxidant status. Addition of antioxidants to cells treated with sOMF arrested apoptosis, whereas hydrogen peroxide generation by the oxidation of benzylamine potentiated GBM cell death. Herein we present a study where we examined effects of sOMF on electron transport chains (ETC), utilizing an O_2_ electrode to measure the consumption of oxygen in isolated mitochondria and on the mitochondria within animal and plant cells. O_2_ consumption experiments in isolated mitochondria have long been used to analyze changes in electron flux in the ETC (19). Using three different, deadly, brain cancer primary cell types we show the mitochondrion is a central target of oncomagnetic therapy and explore the relationship between sOMF, mitochondrial electron flux, and the generation of ROS.

## Materials and Methods

Unless otherwise indicated reagents were sourced from Sigma-Aldrich (St. Louis, MO, USA). Bongkrekic Acid (Cat# 19079) was obtained from Cayman (Cayman Chemical, Ann Arbor, MI, USA) and was dissolved in DMSO and used to a final 0.1% v/v ratio. Mice were obtained from Charles River Laboratories, Inc. (Wilmington, MA, USA).

### O_2_ Electrode Data Collection

**Figure 1:**
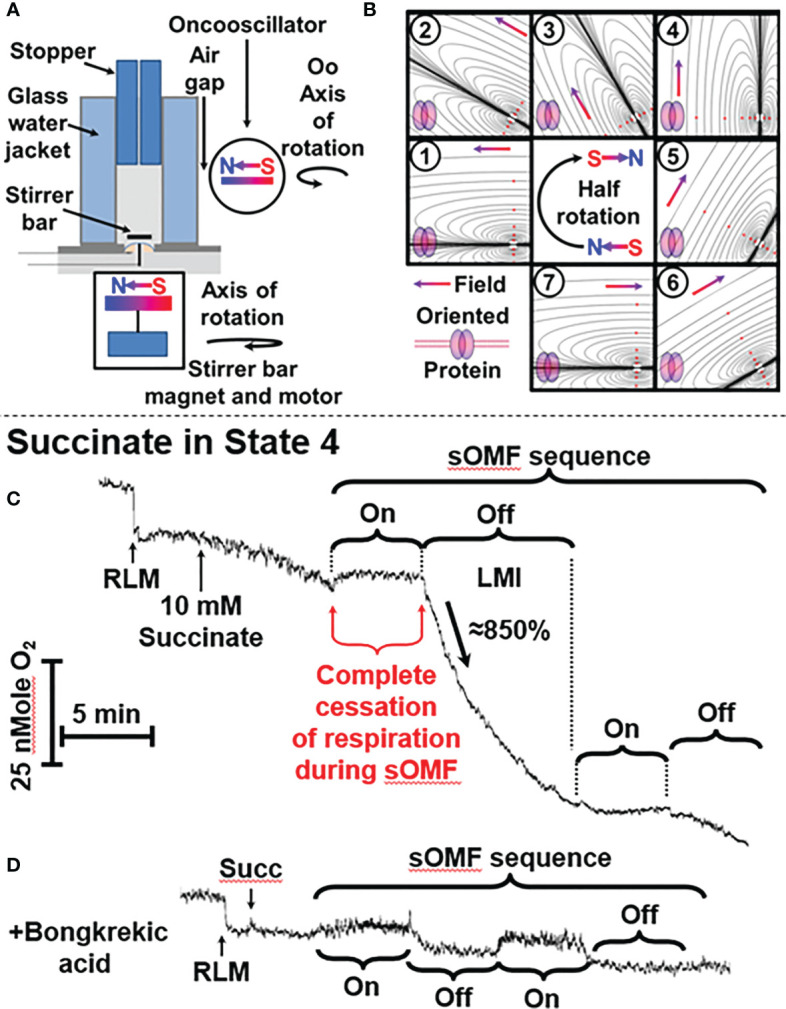
sOMF treatment causes the loss of mitochondrial integrity in State 4 RLM. **(A)** Diagrammatic representation of O_2_ electrode and oncoscillator. **(B)** Diagrammatic representation of the change in the direction of magnetic field orientation and lines of force during a half rotation of an oncoscillator. **(C)** Isolated RLM, respiring on succinate under state 4 conditions, undergo a loss mitochondrial integrity (LMI) after being subjected to 5 min of sOMF stimulation. **(D)** Bongkrekic acid, which blocks the MPT, halts the LMI.

**Figure 2:**
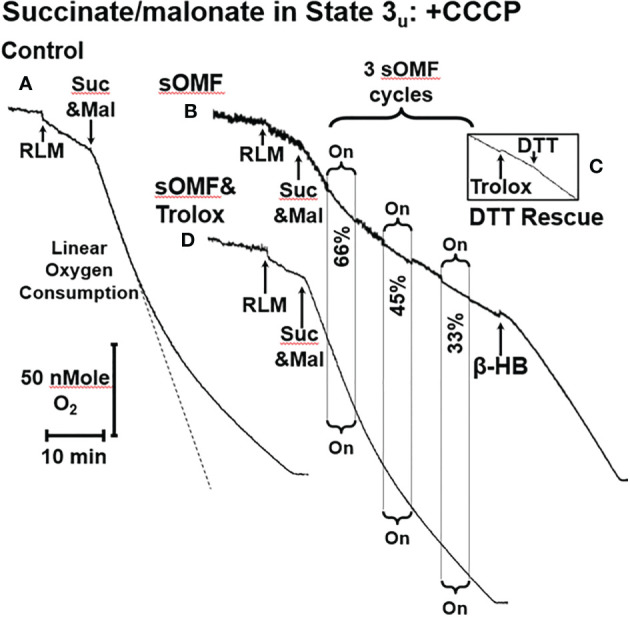
sOMF inhibits SDH in State 3_u_ RLM. **(A)** An O_2_ electrode trace showing the consumption of O_2_ by RLM oxidizing 10 mM succinate in the presence of 500 μM malonate and 2 μM CCCP, State 3_u_. **(B)** sOMF stimulation of under the same conditions causes inhibition of succinate oxidation that continues after halting of field rotation. Addition of β-HB (10 mM) restores mitochondrial respiration showing that the mitochondria have competent complexes I, III and IV. **(C)** An insert of an O_2_ electrode trace from in a parallel experiment to **(B)**, but instead of β-HB, the antioxidant Trolox (15 μM) and DTT (10 mM) was added. Trolox was not able to restore activity to sOMF-treated RLM oxidizing succinate, but DTT can partly restore respiration. **(D)** Pretreatment of RLM with the antioxidant Trolox (15 μM) prior to sOMF treatment can halt the inhibition of succinate oxidation.

**Figure 3:**
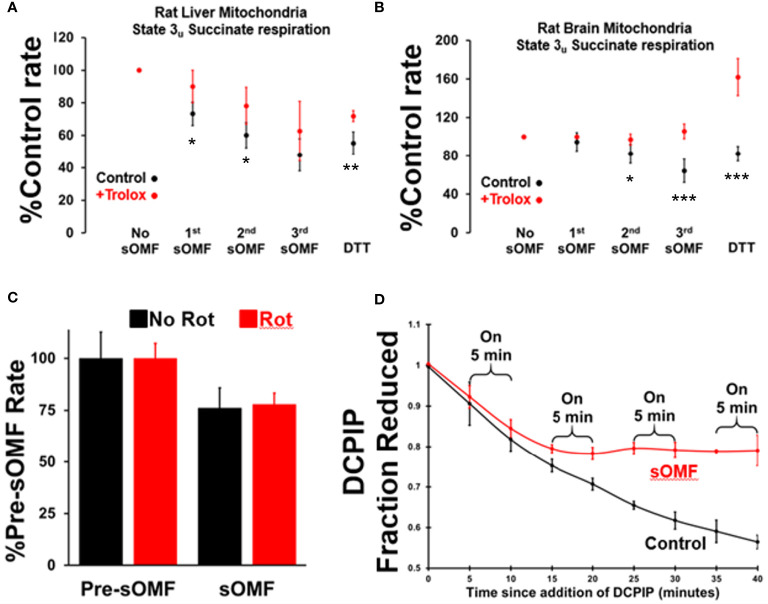
Spinning magnetic fields target SDH in rat liver and brain mitochondria. RLM **(A)** and RBM **(B)** were incubated with/without Trolox (15 μM) and the State 3_u_ (CCCP 2 μM), and respiration was measured with succinate/malonate (10 mM/500 μM). The mitochondrial suspensions were subject to three, 3-minute on/off sOMF stimulation cycles. Rates were measured sOMF post-treatment and mean ± SD of *n*=4 replicants are presented. Noticeable is the almost complete protection afforded to brain mitochondria by Trolox, and that addition of 10 mM DTT boosts the respiration rate above that seen prior to sOMF treatment. *t*-tests were performed and symbols *, ** and *** show points where *p*<0.05, *p*<0.01 and *p*<0.005. **(C)** RLM maintained under State 3_u_ with succinate/malonate (10 mM/500 μM), were subjected to 5 minutes sOMF, in the absence/presence of 2.5 μM rotenone. Succinate respiration fell during the sOMF stimulation period by 76 ± 9.8% in the absence of rotenone and by 78 ± 5.4% in its presence (*n*=6, *p*<0.005 w.r.t. control in both cases). **(D)** The time-course of 150 μM DCPIP reduction by solubilized RLM. Each point represents the mean +/- standard deviation of duplicates from two runs.

**Figure 4:**
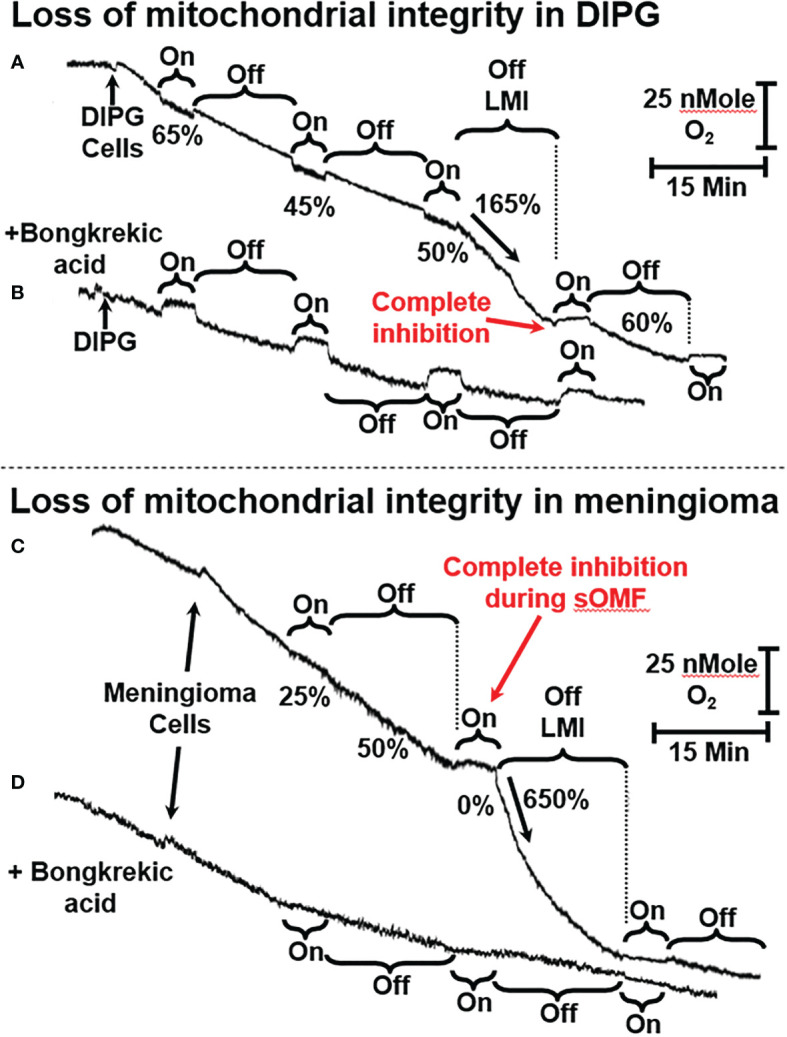
sOMF stimulation of primary DIPG and meningioma cells. **(A)** Mitochondria of primary DIPG cells undergo MPT after repeated cycles of magnetic stimulation, resulting from inhibition of flux and mild uncoupling. **(B)** MPT is blocked by bongkrekic acid in the paired control trace. **(C)** Mitochondria of primary meningioma cells undergo MPT after repeated cycles of magnetic stimulation, resulting from inhibition of flux and mild uncoupling. **(D)** MPT is blocked by bongkrekic acid in the paired control trace.

**Figure 5:**
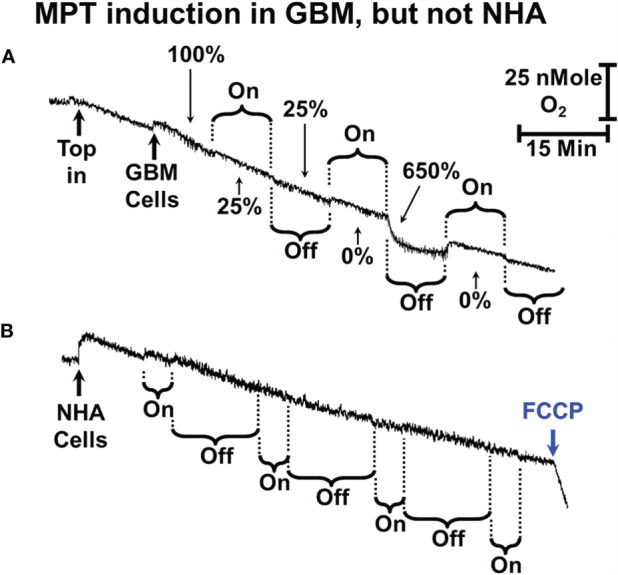
sOMF stimulation of GBM and NHA cells. **(A)** Mitochondria of some primary GBM cultured cells can undergo MPT after repeated cycles of magnetic stimulation. **(B)** Mitochondria of NHA do not undergo MPT with magnetic stimulation. The addition of FCCP at the end of the trace shows that their mitochondria are undamaged and coupled after sOMF stimulation.

**Figure 6:**
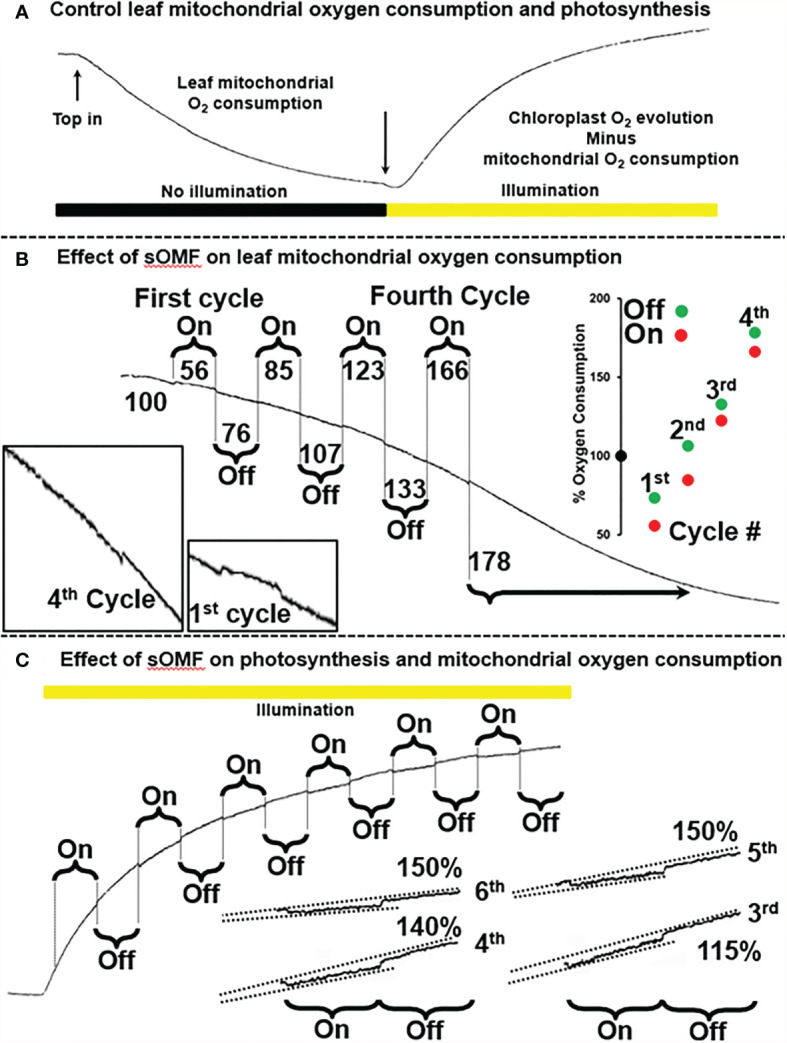
Spinning magnetic field effects on lemon tree leaf. **(A)** A classic O_2_-electrode trace showing the consumption of O_2_ by plant leaf mitochondria, prior to illumination. After illumination photosynthesis is active and the O_2_ levels rise. **(B)** Magnetic stimulation causes a partial inhibition of plant mitochondrial flux as well as mild uncoupling, and these effects are cumulative after repeated cycles of stimulation. **(C)** Magnetic stimulation causes a partial inhibition of photosynthesis.

A thermostatically controlled (37°C), water-jacketed, glass-walled, Clark-type O_2_ electrode (Rank Brothers, Bottisham, Cambridge, UK) was used throughout. The data were collected electronically with a PicoLog^®^ High Resolution Data logger and traces were exported into ImageJ to generate the traces shown in **Figures 1**–**6**. The oncoscillator was attached to the end of a microphone stand and was held perpendicular to the outer glass wall of the electrode, with an airgap of ≈5 mm between the two devices. There was a degree of crosstalk between the stirrer bar of the O_2_ electrode chamber and the oncoscillator; this interaction manifests itself as jumps or drops in the apparent O_2_ concentrations at the beginning or the end of sOMF stimulation. This interaction was found to be at its greatest when the stirrer bar was used at the high speeds typically used to ensure a rapid electrode response rate. The signal to noise ratio differs between O_2_-electrode traces. Before each experiment the stirrer bar, which interacts with the powerful oncoscillator magnet, has to have its velocity adjusted to allow it to have a stable rotation rate.

### sOMF Stimulation

In the O_2_ consumption experiment sOMF stimulation was conducted with an oncoscillator component (magnet-motor assembly encapsulated in a tough plastic tube) of the Oncomagnetic device. The rechargeable battery-operated programmable device controller is triggered by an application on an Android tablet to deliver the desired frequency and timing patterns for stimulation.

For the NMR experiments an sOMF producing oncoscillator array was used. Oncoscillators were mounted on a wooden frame. Repeated intermittent sOMF was applied for 2 hours at a specific frequency, in the 200-300 Hz frequency range, with on-off epochs of 250 or 500 ms duration. Culture plates containing cells were placed on a plastic plate, which in turn was placed on an anti-slip rotating turn table. The turn table was mounted on anti-vibration rubber pads.

### Human Cells

Glioblastoma (GBM) and meningioma cells were from tumors surgically excised from patients by one of the authors (DSB) in 2014 and 2019, respectively. The GBM tumor was a newly diagnosed grade IV cancer obtained from a 29-year-old woman. The meningioma specimen was diagnosed as a grade I tumor from a 41-year-old woman. The DIPG tumor sample, identified as being a K27MH3 mutant, is from an adolescent (17 years old), post-autopsy in 2019. Tumor cells were used under approved Houston Methodist Research Institute (HMRI) Institutional Review Board (IRB) protocol numbers 00014547 and 00017329, and all patients signed an informed consent form approved by the IRB. DIPG cells (SF8628) were also obtained from Sigma-Aldrich (St. Louis, MO, USA). Normal human astrocytes (NHA, CC-2565) were obtained from (Walkersville, MD, USA).

### Preparation of Mitochondria

Rat liver mitochondria (RLM) were prepared according to standard methods (Chappell & Hansford) (20) in a medium containing 250 mM sucrose, 10 mM KPi buffer, 25mM K-HEPES buffer, pH 7.4, 37°C. The mitochondrial protein was assayed using the Pierce™ BCA Protein Assay Kit with bovine serum albumin as a standard. Sodium succinate (10 mM) was added as substrate. 2 µM Carbonyl cyanide m-chlorophenyl hydrazine (CCCP) or carbonyl cyanide trifluoromethoxyphenylhydrazone (FCCP) was used when mitochondria/cells were required in the uncoupled state.

### Preparation of Solubilized RLM for DCPIP/PMS Assay

RLM were frozen/thawed and then incubated for 15 minutes with 10 mM succinate and 5 mM DTT at ≈30°C, to displace oxaloacetate from the succinate pocket (21) and to remove the mixed disulfides (22), respectively. The RLM were pelleted at 5,000 g, washed twice and then dispersed in PBS with 0.1% lauryl maltose (n-Dodecyl β-D-maltoside) detergent, pH 7.4, spun at 15,000g and kept on ice.

### Succinate Dehydrogenase (SDH) DCPIP/PMS Assay

We assayed detergent solubilized SDH activity using a modification of the DCPIP/PMS assay with paired samples, substituting 0.015% lauryl maltoside for 0.1% Triton X-100 (23). As placing rotating magnetic stimulators alongside a cuvette, within a spectrophotometer, could cause sOMF field distortion and interference to electronic components we therefore utilized parallel incubations. Control and sOMF treated paired samples were prepared from a common reaction mixture, from which we removed aliquots, for spectroscopic assay.

Effect of rotenone: PBS supplemented with 0.015% lauryl maltoside, 1 mM sodium cyanide and 2 μg/ml rotenone was maintained at 37°C. A pre-assay incubation mixture of activated SDH was prepared by transferring 5 ml into a centrifuge tube and 10 mM succinate and 1 mg/ml of detergent solubilized RLM was added, at 37°C. After 10 minutes incubation phenazine methosulphate (PMS) and dichlorophenolindophenol (DCPIP) were added to a final concentration of 150 μM from freshly prepared DMSO-stock. The mixture was split into a pair of glass-walled, thermostatically controlled, 37°C, incubation chambers (Rank Bros O_2_-electrode). Two 100 μl aliquots (duplicates) were removed from each chamber, at *t*=0. These aliquots were transferred onto 4-wells of a 96-well-plate and the 600 – 500 nm absorbance was immediately measured using a BioTek Synergy spectrophotometer. At *t*=5 min, the next paired duplicate samples were removed, and a four 5 min on and 5 min off sOMF cycle was applied to the assay mixture in one chamber. Duplicate samples were removed from each chamber at the end of each on/off cycle.

### Tumor and Control Cell Preparations for O_2_-Electrode Experiments

GBM and meningioma tumors were harvested at the time of (first) excision, washed in phosphate-buffered saline (PBS, Fisher Scientific, Waltham, MA), minced with a scalpel, and homogenized in a BeadBug™ homogenizer (Benchmark Scientific, Inc. Edison, NJ) using 1.5 mm Zirconium beads, in an equal volume of PBS. The homogenates were grown in Dulbecco’s modified Eagle’s medium (DMEM) with fetal bovine serum (FBS, 20%), 1U GlutaMax™, sodium pyruvate (1mM), penicillin (100U/ml), and streptomycin (100mg/ml). All cells were expanded from a single T-25 into a pair of T-75s and the DIPG and meningioma cells were used at this passage. GBM cells were grown from frozen stocks of third passage cells and used after the first expansion. NHA were grown in astrocyte cell basal medium supplemented with 3% FBS and the contents of Lonza Clonetics™ AGM^TM^ BulletKit (CC-3186). Prior to O_2_-electrode experiments cells were grown to confluency, counted, harvested using trypsin, washed in ice-cold PBS and stored on ice; then O_2_ consumption of cells was measured in PBS, 10 mM glucose, 37°C.

### Plant Cells

Lemon tree leaves were harvested and chopped into <0.5 mm squares, before being placed in PBS supplemented with 35 mM NaHCO_3_. The leaf fragments were illuminated with a ‘cool’ white light source, a Series 180 Fiber-light^®^ high-intensity illuminator, (Dolan-Jenner Industries, Boxborough, MA, USA), *via* an IR absorbing fiber optic.

### Analysis of Cell Morphology

Cells on coverslips were placed in media containing 500 nM MitoTracker™ for 30 min. Then they were transferred into a temperature-controlled perfusion chamber and maintained at 37°C with a Warner Instrument Corp (Hamden, CT) TC-344B temperature controller. Cells were imaged using a Nikon Eclipse TE2000-E at 60× or 90× magnification with a CoolSnap ES digital camera system (Roper Scientific). Image acquisition and analysis was done using Nikon NIS-Elements software.

### Optical Monitoring of Serum-Starved GBM Cells

To study sOMF effects on non-dividing GBM cells, after 24 hours of fetal bovine serum (FBS) starvation GBM cells were transferred into 20% FBS media. Changes in mitochondria were observed in GBM cells loaded with 500 nM MitoTracker™ and then sOMF or SHAM stimulated cultures were observed over 60 min using time-lapse video fluorescence microscopy.

Bright field time-lapse video microscopy was used to observe morphological changes characteristic of cell death in these serum-starved GBM cells. In parallel cultures, cells were pre-incubated with 10 μM CellEvent™ Caspase-3/Green (Invitrogen™) and an image taken at 1 hour, near the peak of caspase-signaling is shown.

It should be noted that serum-starved GBM cultures are much more sensitive to sOMF than those grown and maintained with FBS.

### Observation of Caspase3/7 Activation in GBM

GBM cells were grown in Petri dishes with 20% FBS media. Confluent cells were loaded with 5 μl/ml NucView Caspase-3/7 Green Detection Reagent (Biotium, San Francisco, USA) for 1 hour, washed with pre-warmed PBS, and then reimmersed in media. The dish was transferred to the thermostatically controlled microscope stage and time-lapse fluorescence images were acquired every 10 minutes. After the first image was recorded, the cells underwent sOMF for 2 hours, and images of post-treatment apoptosis were acquired for a further 14 hours. A typical movie of caspase 3/7 activation in GBM cells treated with sOMF, hours after elevation of mitochondrial superoxide generation, is to be found in our prior paper (2).

### ^1^H and ^13^C NMR Experiments and Isotopomer Analysis

^1^H and ^13^C NMR data were acquired on a Bruker NMR spectrometer (Bruker Biospin, Billerica, MA) operating at 800 MHz (^1^H frequency) or 201 MHz (^13^C frequency), equipped with a cryogenically cooled ^13^C direct detection probe.

SF8628 patient derived DIPG cells were grown in high glucose (25 mM) Dulbecco’s modified Eagle’s medium (DMEM) supplemented with 10% fetal bovine serum (FBS), 2.0 mM glutamine at 37°C under humidified air with 5% CO_2_. Cells were divided into 2 groups (*n*=3 in each group): With sOMF-treatment (Mag) and without sOMF treatment (Sham). Cells in all replicates were cultured in square culture dishes (245 mm × 245 mm) until confluency was reached (~5.0×10^6^ cells/mL). During the final 2 h, cells from both groups (Mag and Sham) were treated with 11.0 mM of [U-13C]glucose (Millipore Sigma, Miamisburg, OH) in DMEM (supplemented with 10% FBS, 2.0 mM glutamine). After 2 hours of magnetic or sham stimulation, the medium was removed by aspiration, cells were washed with cold PBS buffer, trypsinized, cell pellets were snap-frozen in liquid nitrogen, and were stored at -80°C until further analysis by ^1^H/^13^C NMR spectroscopy. Cell pellets were extracted in 5% perchloric acid, centrifuged to remove the cellular debris, neutralized, and dried in a CentriVap^®^ vacuum concentrator (Labconco Corporation, Kansas City, MO). Residues were reconstituted in 180 µL D_2_O containing 1.0 mM DSS-*d6* (as an internal standard), pH was adjusted to 7.4. For obtaining ^1^H NMR spectral data 90° excitation pulse with a duration of 10 µs was used with 128 scans. Number of points in the time domain were 132 k and the relaxation delay was 2.0 s. The spectral width was 9,615 Hz and the acquisition time was 1.70 s. The nuclear Overhauser effect mixing time used was 100 ms. ^1^H NMR time domain data were Fourier transformed by applying an exponential window function with a line broadening of 0.3 Hz. The resulting frequency domain spectrum was referenced to the internal standard DSS-*d6* (0 ppm).

For ^1^H-decoupled ^13^C NMR experiments, a power-gated sequence with a WALTZ-16 composite pulse (flip-angle = 30°) was used. A 90° pulse with a duration of 10 µs was used with 10,000 scans. Number of points in the time domain were 144 k and the relaxation delay was 2.5 s. The spectral width was 36,058 Hz and the acquisition time was 2.0 s. Time domain data were processed by applying an exponential window function with line broadening of 1.0 Hz. The lactate C3 carbon signal at 20.8 ppm was used as an internal chemical shift reference. Peak areas of various ^13^C signals were determined by deconvolution using the ACD software (Advanced Chemistry Development, Toronto, Canada). ^13^C NMR isotopomer analysis was performed as reported in our earlier publications (24, 25).

## Results

We used an apparatus consisting of an oncoscillator in all investigations of O_2_ consumption/generation. This apparatus generates sOMF by rotating a permanent magnet with a high-speed direct current electric motor. The oncoscillator assembly was held by a microphone stand, placed next to the water jacketed O_2_ electrode with a 5 mm air gap, preventing vibration transfer. The oncoscillator was positioned so that the center of 1 ml media chamber is exposed to a magnetic field of ≈80 mT (**Figure 1A**). The rotation of the oncoscillator magnet sometimes causes a small artifactual effect on the O_2_ electrode stirrer bar giving rise to an apparent drop or rise in O_2_ levels that can be distinguished from the sOMF effect.

The range of potential magnet rotation frequencies and acceleration/deceleration of the resulting waveforms is vast, and we have only explored a limited range of the options available. The peak nominal sOMF frequency used was ≈280 Hz (wave duration of ≈3 ms), although we have explored a wide range of frequencies between 50 and 350 Hz. The oncoscillator was turned on for 250 ms and then turned off for 250 ms, allowing the oscillations to slow down. During this on/off power cycle the oncoscillator performs a rapid sweep through increasing frequencies (during acceleration) and a slower sweep through decreasing frequencies (during deceleration). The frequency sweeps quite possibly span the cycling rates of electron fluxes associated with different mitochondrial states (26, 27). Short 5 to 15 min runs of on-off cycles were separated by pauses of similar durations. These values were derived by testing a wide range of timings using a variety of cells. A visual representation of the change of field orientation, with respect to an idealized membrane protein, is shown in **Figure 1B**. The magnetic field polarity and directionality is indicated by the south-north, red-blue gradient colored arrow and idealized lines of force are also shown. A half-rotation, numbered from (1) to (7), begins from an initial field parallel position, and proceeds into the perpendicular (4), and returns to parallel orientation, but with the field having an opposing polarity (7). This field rotation will allow all spins in the x-y orientation to find themselves aligned with the external field. This process is facilitated further by the stirring of the mitochondria/cells within the chamber by the magnetic stirrer along the z-axis domain. Thus the proteins within the cells are undergoing orientation changes whilst treated with a rotating field.

### Rat Liver Mitochondria

We highlight the effects of sOMF on succinate oxidation by rat liver mitochondria (RLM) as RLM are the most studied mammalian mitochondria with a wealth of knowledge now available on their functional characterization (28, 29). SDH, Complex II of the mitochondrial respiratory chain is well characterized and the mechanisms by which it generates ROS, in the form of superoxide and hydrogen peroxide, are well studied (30). Known sites of ROS generation in mitochondria undergoing succinate respiration include the flavin radical of SDH and also Complex I, which is due to reversed electron transport. This ROS generation has been shown to be driven, in part, by elevated mitochondrial membrane chemiosmotic potential (ΔΨ) and ubiquinol (QH_2_) (31–35). We present the effects of sOMF on mitochondrial O_2_ consumption, utilizing succinate as substrate in the presence and absence (induced by carbonyl cyanide m-chlorophenyl hydrazone, CCCP) of a chemiosmotic potential, i.e. mitochondrial states 4 and 3_u_, respectively.

### Loss of Mitochondrial Integrity in RLM in State 4

**Figure 1C** shows a typical O_2_ electrode trace showing the effects of 5 min of sOMF stimulation on mitochondrial respiration, i. e. RLM oxidation of succinate. After succinate addition, the state 4 RLM have the typical slow rate of O_2_ consumption, ≈10 e^-^ s^−1^ (36–38). Electron flux through SDH, and Complexes III and IV is slowed by product inhibition, due to the presence of a large membrane potential (ΔΨ) and ΔpH, measured as 183 mV and 11 mV, respectively, by Brown and Brand under similar conditions (28).

When sOMF stimulation is initiated, mitochondrial respiration is completely halted for the whole 5-min sOMF stimulation period. Immediately after cessation of field rotation we observe a loss of mitochondrial integrity (labeled LMI), with a very rapid increase in O_2_ consumption, initially ≈ 86 e- s^−1^, followed by first-order type fall in oxygen consumption. After 10 min, sOMF stimulation is restarted, and a complete inhibition of respiration can again be observed. Following termination of the second magnetic stimulation regime, respiration restarts at a similar rate to that observed prior to the stimulation cycle.

The line-shape of respiration rate after cessation of sOMF, (labeled LMI), is what would be expected if the mitochondrial permeability transition (MPT) (39, 40) were to be universally and simultaneously triggered. We therefore examined the effects of pre-incubation of RLM with a MPT inhibitor under similar conditions. Bongkrekic acid is a specific inhibitor of the adenine nucleotide translocase (ANT) and blocks the MPT in isolated mitochondria or cells (39, 41, 42). **Figure 1D** shows RLM undergoing sOMF stimulation, but here pre-incubated with 5 µM bongkrekic acid. The RLM in this negative control do not suffer from loss of integrity, which is evidence for the induction of the MPT by sOMF (**Figure 1C**). We chose bongkrekic acid over cyclosporin A (CsA) because CsA can have profound effects on cancer cells, especially with respect to cell metabolism and in reshaping their antioxidant defenses. CsA has been shown to induce apoptosis of glioma cells, by a direct increase in the levels of p53 (43).

In a paper on non-small cell lung cancer (44) Qin and Chen noted that low concentrations of CsA increased the phosphorylation of Akt, increased expression of Cyclin D1, and decreased expression of p27. They also noted that these low levels of CsA elevated the level of ROS. Yu et al. (45) also noted that CsA suppresses ROS-mediated translocation of p53 from the cytosol into the mitochondria, where mt-p53 aids the opening of the mitochondrial permeability transition. As p53 has a central role in most cancer cells’ balance between viability and death, we did not want to see a cancer-cell specific effect of CsA, rather than an inhibition of the MPT. Many researchers prefer bongkrekic acid over CsA, because it only has a single target, the ANT, and works in both isolated mitochondria and in cells. However, we repeated the State 4 RLM experiment shown in **Figure 1D** by substituting 0.5 μM CsA for 5 μM bongkrekic acid. In this experiment we observe essentially the same, flat, respiration of the RLM with succinate as substrate, with no LMI observed.

### SDH Inhibition by sOMF in State 3_u_ RLM Is Caused by ROS Generation

State 3u is the name given to state of mitochondria incubated with both uncoupling agent and a substrate (46). Quinlan and co-workers showed coincubation of mitochondria with succinate (10 mM) and competitive inhibitor malonate (0.4-1 mM) produces negligible ROS (23, 33). We sought to unambiguously demonstrate that sOMF affect discrete electron states of SDH, and that in these states ROS is generated, and this is due to the action of the imposed sOMF. Therefore, we examined the oxidation of succinate (10 mM) in the presence of malonate (0.5 mM) in uncoupled (CCCP-treated) RLM.

**Figure 2A** shows a control trace of CCCP-treated, malonate-inhibited RLM oxidizing succinate, conditions which give rise to succinate oxidation at a similar rate as ΔΨ-inhibited RLM in state 4, shown in **Figure 1C**. **Figure 2B** shows the effects of 3-min on/3-min off sOMF treatment cycles on succinate oxidation under the same conditions. Repeated treatments are chosen to highlight the increasing inhibition of succinate oxidation induced by sOMF treatment. During the first cycle of sOMF the rate of O_2_ consumption falls to ≈66% of the initial rate. After cessation of field rotation, the oxidation rate neither recovers nor falls. When subjected to two more cycles of sOMF the rate of O_2_ consumptions falls to 45% and then to 33%, with no recovery in respiration after stopping sOMF stimulation. To be sure that SDH, and not Complexes III or IV was the site of sOMF inhibition, we added β-hydroxy-butyrate (β-HB, 10 mM) to bypass SDH. β-HB metabolism generates Complex I, III and IV respiratory substrates and allows mitochondrial flux in the absence of functional SDH. β-HB greatly increased the rate of O_2_ consumption, suggesting the SDH is more likely to be the primary site of sOMF-induced inhibition of mitochondrial respiration. The insert **Figure 2C** shows a portion of an O_2_-electrode trace from an experiment identical to that shown in **Figure 2B**, except that instead of β-HB, Trolox was added, followed by dithiothreitol (DTT, 10 mM). Trolox does not elevate the rate of succinate respiration in sOMF-treated mitochondria, but addition of DTT restores half of the SDH activity. Thus, sOMF inhibition of SDH activity can be partly restored using the membrane permeant thiophilic reducing agent, DTT.

We have previously demonstrated that sOMF treatment of cells generates superoxide/hydrogen peroxide in the mitochondrial matrix (2), and SDH is a site of mitochondrial superoxide generation (23, 33). To test if the post-sOMF observed inhibition of SDH is mediated by ROS generated by the action of sOMF on SDH, we repeated the same experiment in the presence of Trolox, which protects thiols from ROS oxidation (47). sOMF treatment of RLM in State 3_u_ pre-treated with Trolox (15 μM), show minimal inhibition, as can be seen by comparing **Figures 2B, D**, with respiration almost impervious to sOMF. This effect of Trolox, preserving SDH activity when stimulated by sOMF, may be the mechanism by which we were able to maintain cancer cell viability after sOMF therapy with this antioxidant (2).

We carried out a systematic study on the protective effects of Trolox on the inhibition of succinate respiration, in state 3_u_ conditions, utilizing both rat liver and rat brain mitochondria (RBM). Mitochondria were preincubated with or without 15 μM Trolox and then underwent three 3-min on/off sOMF cycles. Finally, DTT was added to assess the fraction of recoverable activity. In **Figure 3** we show plots derived from 4 repetitions of this experiment, for the post sOMF rates observed in both RLM (A.) and RBM (B.), with statistical significance calculated using the t-test. Succinate respiration clearly undergoes ROS-dependent inhibition post-sOMF, with Trolox being protective in both mitochondrial preparations, essentially blocking all inhibition in brain mitochondria. Increases in respiration following addition of DTT, suggest that thiol oxidation in SDH may result from sOMF.

It is possible that the inhibition of State 3_u_ respiration we demonstrate in **Figures 3A, B**, may be artifactual, caused by the generation of the SDH-inhibitor oxaloacetate. As the Complex I inhibitor rotenone stops NADH oxidation, halting oxaloacetate generation during succinate oxidation, we examined the impact this agent had on sOMF inhibition of succinate respiration. In **Figure 3C** we show inhibition of RLM, maintained under State 3u with succinate/malonate prior to and during 5 min sOMF, in the absence and presence of 2.5 μM rotenone. Succinate respiration fell during the sOMF stimulation period by 76 ± 9.8% in the absence of rotenone and by 78 ± 5.4% in its presence (*n*=6, p<0.005 with respect to control in both cases). There is no statistically significant difference between the level of sOMF inhibition of State 3u due to the presence of rotenone.

To be sure that SDH was inhibited by sOMF we examined the reduction of DCPIP by solubilized RLM with succinate as reductant (23). **Figure 3D** shows that SDH is a target of sOMF, and that inhibition of activity lingers after sOMF treatment. In this assay we were able to abolish all SDH activity after only two 5-min sOMF cycles. The differential sOMF sensitivity of State 3u versus detergent solubilized SDH suggests that mitochondrial matrix mechanisms are responsible for protecting SDH from sOMF-generated ROS.

The persistent, Trolox-sensitive, inhibition of SDH by sOMF can be explained by the presence of redox sensing thiol at the succinate/flavin adenine dinucleotide (FAD) binding site of SDH. Treatment of SDH with thiophilic reagents like N-ethylmaleimide (NEM) inhibit succinate flux, with the reactivity toward NEM blocked by incubating the enzyme with substrates or inhibitors that occupy the substrate binding pocket (48). SDH generates superoxide/hydrogen peroxide (33) and these oxidants cause enzyme inhibition due to oxidation of Cys90 and formation of a glutathionyl-mixed disulfide (22). Trolox can eliminate superoxide and hydrogen peroxide (47), but cannot reduce protein-glutathione disulfides. **Figures 2** and **3** show that while Trolox can intercept SDH/sOMF-derived inhibitory oxidants, it cannot repair deactivated enzyme, whereas DTT has some repair function. Glutathione in the mitochondrial matrix can provide some protection from ROS, but after solubilizing the mitochondria, this protection is lost and the SDH becomes more sensitive to sOMF.

### The Mitochondrial Permeability Transition in Tumor Cells and Astrocytes

In **Figures 4** and **5** we present representative O_2_ electrode traces of the effects of sOMF stimulation on primary cultures of three brain tumor cell types, namely DIPG with the histone H3K27M mutation, meningioma, and GBM. NHA were used as a control. **Figure 4A** trace shows the effect of sOMF stimulation on 2 × 10^6^/ml DIPG cells in glucose-supplemented phosphate-buffered saline (PBS). The DIPG cells exhibit a high respiratory flux, typical of the H3K27M phenotype (49). Before sOMF stimulation the O_2_ consumption rate is ≈40 pMol O_2_ s^-1^ million cells^-1^. But during the first 5-min sOMF stimulation cycle respiration falls by a third and during the first 15-min pause this respiration rate remains at 65% the initial rate. During the second stimulation cycle respiration falls to only 45% of the control rate. When the cycle ends the rate rises to 58%, but over the 15-min pause the flux slows down. During the third sOMF cycle O_2_ consumption is held at half the initial rate, but when rotation ceases, we observe a rapid increase in respiration, labeled as LMI. Finally, the fourth stimulation cycle completely abolishes respiration and when the stimulation ceases the rate is at first 60% of the initial rate and then falls off.

The large increase in mitochondrial electron flux that is observed at the end of the third sOMF cycle is consistent with a homogenous population of cells undergoing the MPT simultaneously. To support this interpretation of the data, we repeated the experiment in the presence of 5 µM bongkrekic acid (**Figure 4B**). Blocking the ANT with bongkrekic acid slows the steady state mitochondrial electron flux to 55% of that seen in its absence (both rates are normalized to 100% in each panel for ease of interpretation). Under this condition we observe that respiration is slowed during sOMF stimulation but there is no increase in rate indicative of the permeability transition.

In **Figures 4C, D** we show that sOMF stimulation in primary meningioma cells also causes the LMI. In these cells there is a 75% drop in the electron flux, and after the end of the first sOMF cycle, the cells maintain only half their initial rate (**Figure 4C**). During the second cycle of sOMF mitochondrial respiration is completely halted, and as soon as rotation stops there is a burst of O_2_ uptake that is >6-fold the initial rate, labeled LMI. Again, the LMI feature is abolished by pretreating cells with MPT-blocking agent, bongkrekic acid (**Figure 4D**).

We also examined the effect of sOMF stimulation cycles on the O_2_ consumption rate of a primary GBM cell line (GBM157) (**Figure 5A**). In cultured GBM157 cells we used 10 min stimulation/pause cycles to cause the LMI. The first stimulation drops the mitochondrial flux by 75%, with no recovery following cessation of stimulation. The second stimulation slows the rate to zero, but immediately after the stimulation stopped, we observed a burst of respiration, followed by a slowing, again with a first order line shape.

We performed a variety of combinations of stimulation/pause cycle durations in NHA but were unable to cause either inhibition of oxygen consumption or a rapid uptake of oxygen indicating uncoupling/LMI. A multicycle trace exemplifying this is shown in **Figure 5B**. Here we used 5-min stimulation followed by a 15-min recovery period and ran 4 cycles before adding FCCP. Over the 4 cycles respiration dropped by approximately 50%, but the cells maintained their controlled respiration, as is seen by the accelerated rate of O_2_ consumption after the addition of uncoupler trifluoromethoxy carbonylcyanide phenylhydrazone (FCCP).

The lack of major effects in NHA here are very similar to the studies we previously carried out on viability of NHA after sOMF treatment, where we observed no statistically significant effects (2).

### Plant Mitochondria and Photosynthesis

Next, to investigate the generalizability of the effect of sOMF on mitochondrial redox processes, we studied the effect of sOMF on lemon tree leaf, in the absence and presence of illumination, especially, given that effect of magnetic stimulation on photosynthesis through RPM have been documented (7, 8) (**Figure 6**). **Figure 6A** shows a control trace of lemon tree leaf first consuming O_2_ in the absence of illumination, and then generating O_2_ *via* photosynthesis after illumination. In **Figure 6A**, we see the effects of four cycles of sOMF stimulation on leaf O_2_ consumption, with the effects of the first and fourth cycles highlighted. The numbers above and below the trace indicate the O_2_ consumption rate before, during and following a magnetic stimulation cycle. During the first 5-min magnetic stimulation cycle, we observe a drop in the respiration rate and, after cessation of stimulation, an increase that comes to a steady state at 85% of the initial rate. Subsequent cycles have a similar pattern of inhibition/activation, with the final rate, after four cycles, being almost double the initial rate. The best explanation is that sOMF stimulation inhibits electron flux and causes mild uncoupling of the plant mitochondria.

After allowing the O_2_ concentration to drop to its lowest steady state of <5 µM, a bright light source was used to activate leaf photosynthesis. After 3 min of illumination the leaf was subjected to six 5-min sOMF stimulation/pause cycles. Blowups of the traces of the last four cycles are shown with added lines representing O_2_ generation rates. If we compare the rate of O_2_ increase during a stimulation cycle with the rate seen immediately afterward it is clear that O_2_ evolution is inhibited. In the case of the fifth and sixth cycles the rate after stimulation is 150% of the rate observed during stimulation.

### sOMF Drives Mitochondrial Initiated Death in DIPG Cancer Cells

**Figure 7:**
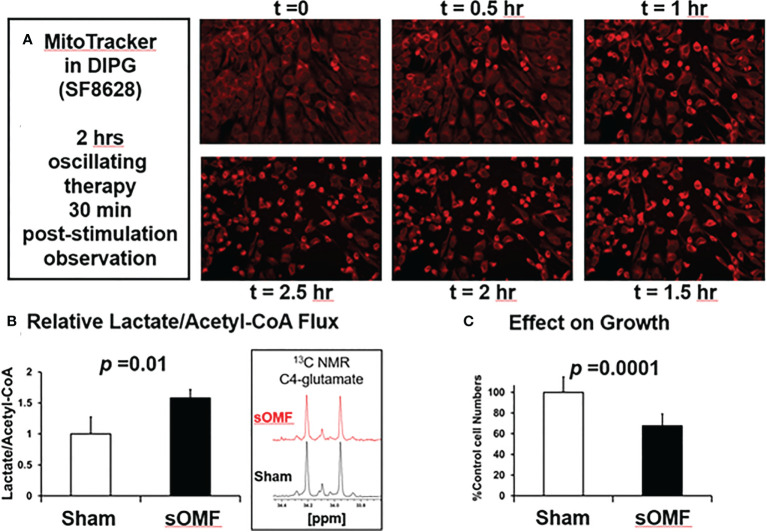
sOMF treatment of SF8628 DIPG cells causes cell death and mitochondrial changes. **(A)** Effects of sOMF in paired sOMF/sham-treated SF8628 DIPG cells, preincubated with MitoTracker™. Fluorescence microscopic images, taken every 30 min during two hours of sOMF treatment and at 30 min post-treatment show classical morphological changes indicative of cell death, which were not observed in control cells (data not shown). **(B)** ^13^C-glucose metabolism studies show that 4-h sOMF stimulation elevates anerobic/aerobic flux of DIPG cell cultures by ≈60% (*n* = 3, *p* = 0.01), based on levels of ^13^C-lactate and ^13^C-glutamate (insert). **(C)** Viability dye Hoechst 33342 studies performed 44 hours after 4 h of sOMF or sham stimulation of SF8628 DIPG cells show a ≈40% drop in viable cells (*p*<0.0001) due to sOMF-treatment.

In **Figure 7** we illustrate sOMF-induced changes in cultured SF8628 DIPG cells. Cells loaded with MitoTracker™ were subjected to sOMF stimulation for 2 h, and then observed for a further 30 min, at which time simultaneous cell death was observed (**Figure 7A**). SF8628 cellular metabolism was examined using ^1^H and ^13^C nuclear magnetic resonance (NMR) in cells incubated with ^13^C-glucose, with cells subjected to 4 h of sOMF or sham stimulation. Sham stimulation involved rotation of a non-magnetic rod. We find that sOMF stimulation causes an elevation of flux from ^13^C-glucose into ^13^C-lactate and a decrease in ^13^C-acetyl-CoA. sOMF stimulation results in the mitochondrial carbon flux falling by ≈25% and lactate flux rising by ≈16%. sOMF treatment therefore raises the anerobic/aerobic flux of these DIPG cells by ≈60% (*p*=0.01, **Figure 7B**). In growth studies we examined the effects of 4 h of sOMF or sham stimulation on living SF8628 DIPG cell numbers assayed 44 h later. Using this regime, we observed a ≈40% drop in viable cells, (*p*<0.0001, **Figure 7C**).

### sOMF Effects on Mitochondria and Apoptosis of Cultured, Non-Dividing, Cancer Cells

**Figure 8:**
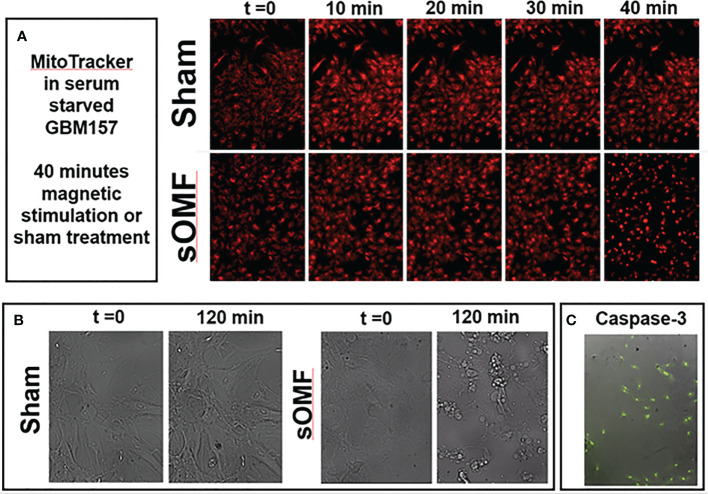
sOMF kills non-dividing GBM cells *via* effects on their mitochondria. **(A)** Serum-starved, non-dividing GBM cells stained with MitoTracker™ and stimulated with sOMF or sham were observed over 40 min by time-lapse video fluorescence microscopy. Image frames at five time points demonstrate that cell death in the sOMF-treated but not sham-treated cultures at 40 min. Paired sOMF **(B)** and sham-treated **(C)** serum-starved cultures, examined using bright field microscopy, also show morphological cell death only with sOMF treatment., and with green fluorescence indicating activation of the caspase-3 apoptotic pathway in sOMF-treated, but not sham-treated (data not shown) cell cultures.

In **Figure 8** we demonstrate that sOMF is highly effective at killing non-dividing GBM cell cultures, examining GBM cells by both fluorescence and white light microscopy. Serum-starved GBM cell cultures are hyper-sensitive to sOMF stimulation wherein cells loaded with MitoTracker™ undergo cell death in 40 min, but sham-treated controls appear to be completely normal (**Figure 8A**). The generation of the reactive singlet O_2_ during fluorescence microscopy appeared to be aiding the toxicity of sOMF stimulation. When paired sOMF/sham serum-starved GBM cultures were examined with bright field microscopy, we were able to observe synchronized cell death in cultures subjected to 2 h of sOMF stimulation, but little effect on sham-treated paired cultures (**Figure 8B**). To be sure that we were observing a mitochondrial-based death cascade we examined the levels of caspase-3 activation 1 h after sOMF/sham treatment of serum-starved GBM cultures. In **Figure 8C**, we show an image of a typical bright field/caspase-3 green fluorescence reporter panel, indicating that the mitochondrial/caspase-3 apoptotic pathway has been triggered by sOMF stimulation. No caspase-3 activation was seen in sham-treated controls (data not shown).

### Activation of Caspase-3/7 in sOMF-Treated GBM Cells Is Concerted

**Figure 9:**
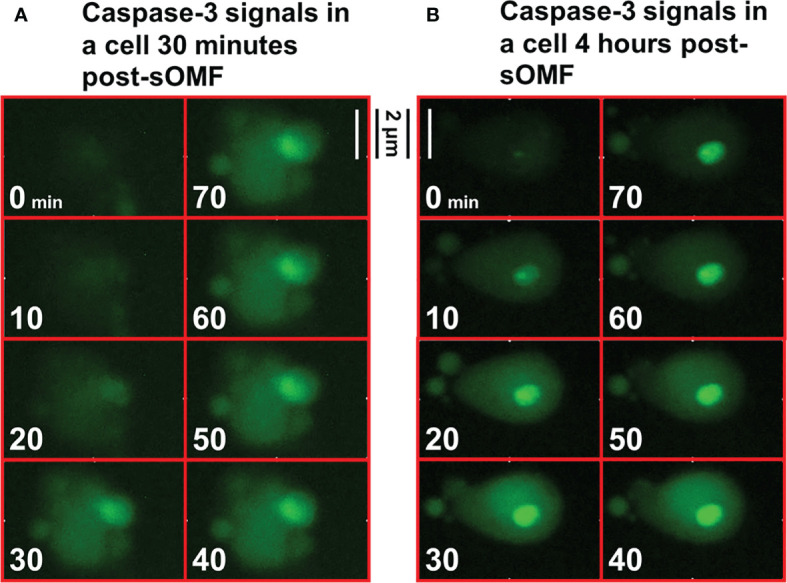
High resolution images of the activation of caspase-3/7 in sOMF treated GBM. GBM cells were loaded with NucView Caspase-3/7 Green, washed, and then returned to media. Time-lapse fluorescence images were acquired every 10 minutes for 16 hours. The cells underwent sOMF for 4 hours, and post-treatment apoptosis was monitored for a further 12 hours. We picked two representative cells that can be observed to undergo rapid caspase-3/7 activation, the first **(A)** some 30 minutes and these second at 4 hours **(B)** post-sOMF therapy. We show movies stills, taken 10 minutes apart, to show the time dependency of caspase-3/7 activation in these GBM cells.

Cultured GBM cells loaded with a caspase-3/7 fluorescence reporter, then underwent sOMF for 4 hours, and were monitored for a further 12 hours. **Figure 9** shows high resolution images of two cells undergoing rapid activation of caspase-3/7. The first series of images (9A) begin 30 minutes after the end of sOMF stimulation (*t*=0), and the second (9B) some 4 hours post-sOMF therapy. In each case it can be observed that caspase-3/7 occures rapidly, and suddenly, within the entire cytosol of the cell. This suggests that there is an event within each sOMF-treated cell that causes near simultaneous MPT, release of cytochrome *c* and other apoptosis-inducing factors, resulting in caspase-3/7 activation in these GBM cells.

## Discussion

Magnetic fields as weak as that of the Earth (≈50 µT) can alter the kinetics of RPM-based reactions, and such systems are used biologically for navigation in migrating birds (50). The effects of weak µT and mT range magnetic fields on chemical reactions are not powerful enough to exert thermodynamic control on biological processes but are instead determined by electron spin selection rules (14).

**Figure 10:**
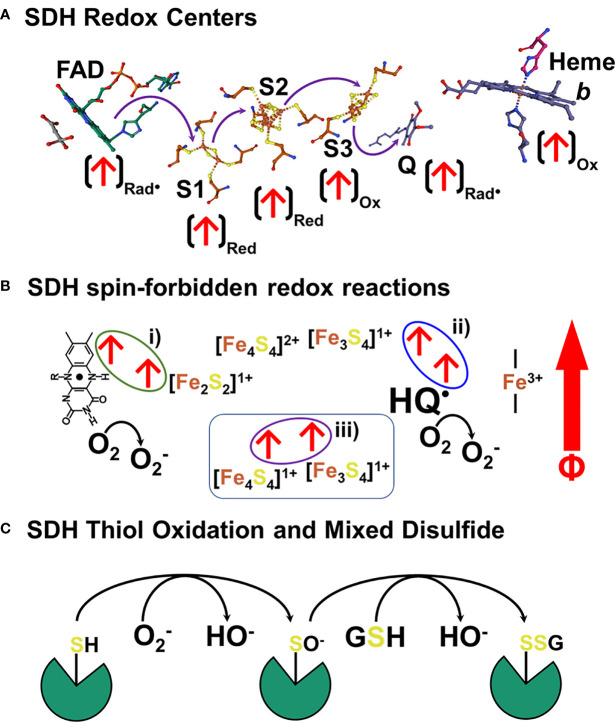
Schematic representation of a hypothetical mechanism of SDH inhibition and ROS generation in response to sOMF. **(A)** shows the arrangement of redox centers in SDH, from the substrate binding site (left) occupied by oxaloacetate to the heme *b* (right), based on the 1YQ3 crystal structure. An arrow at each redox center, (↑), identifies the redox state when the center is a radical (ox, red or rad•). There are three different biradical pairs that may be subject to coupling to an external magnetic field. Firstly, the flavin radical and the S1 [Fe_2_S_2_]^1+^ center, 10B i). Secondly the biradical-redox pairing of S2 [Fe_4_S_4_]^1+^ and the S3 [Fe_3_S_4_]^1+^ center, **(B ii)**. Finally, the S3 [Fe_3_S_4_]^1+^ semiquinone radical (HQ•) radical pair, **(B i)**. Trapped flavin and quinone radicals, could reduce paramagnetic oxygen to superoxide. In **(C)** we present a simple scheme that shows how SDH is inactivated by sOMF, based on SDH thiol sensing, with sOMF treatment causing SDH to generate superoxide/peroxide. Either superoxide or hydrogen peroxide oxidizes a controlling thiol of SDH to the sulfenic acid. The cystenic acid would then react with glutathione (GSH) and cause the formation of the SDH-glutathionyl mixed disulphide, blocking the substrate pocket.

We have demonstrated that sOMF markedly alter mitochondrial oxidative processes, in isolated mitochondria and in human cancer and plant cells. Our focus here has been on the inhibition of succinate flux, in state 4, state 3_u_ and using detergent solubilized RLM to showcase our technology on the simplest of ETC complexes, SDH. In **Figure 10** we present a model for the action of sOMF on SDH, based on the data presented herein. **Figure 10A** shows the arrangement of redox centers in SDH, from the substrate binding site (left) occupied by oxaloacetate to the heme *b* (right), based on the avian respiratory complex II structure with oxaloacetate and ubiquinone (1YQ3) from Ed Berry’s group (51). Each of the redox centers which generates a radical during catalysis is identified by an arrow (↑), as is the redox state when the center is a radical (ox, red or rad•). The heme of SDH is not redox active in the avian/mammalian SDH enzyme when oxidizing succinate (52) and is ignored in this narrative. Oxidation of succinate by the flavin in the substrate pocket is a two-electron process, and the passage of single electrons along the three [FeS] centers (S1, S2 and S3) to the ubiquinone gives rise to three different biradical pairs that may be subject to coupling to an external magnetic field. Oxidation of the flavin ring by the S1 [Fe_2_S_2_]^2+^ generates a biradical pair consisting of the flavin radical and the [Fe_2_S_2_]^1+^ center, shown in **Figure 10B** i). Reduction of the S2 [Fe_4_S_4_]^2+^ by S1 forms a second biradical-redox pairing S2 [Fe_4_S_4_]^1+^ and S3 [Fe_3_S_4_]^1+^, shown in **Figure 10B** ii). Finally, when reduced S3 is oxidized by quinone it generates the S3 [Fe_3_S_4_]^1+^ semiquinone radical (HQ^•^) pair, shown in **Figure 10B** ii). If any of these redox electron pairs become captured and have a spin orientation affected by an externally applied magnetic field, then they may be trapped in a spin-forbidden state, inhibiting electron passage, and therefore slowing enzyme flux. Two of these sites capable of being trapped in a spin-forbidden state, the flavin and quinone radicals, could reduce paramagnetic O_2_ to superoxide (**Figure 10B**).

The generation of ROS by mitochondria is an inevitable feature of electron transfer in an O_2_ rich environment, and so multiple feedback loops exist to sense ROS levels and lower ROS generation. The best-known ROS-sensing/ROS-reducing feedback loops are the aconitase/Krebs cycle loop and Phospholipase A2/uncoupling protein loop. Superoxide inhibits aconitase, slowing the Krebs cycle, lowers the NADH/NAD^+^ ratio, and so slows electron entry into the mitochondrial ETC (53–55). Superoxide activates mitochondrial phospholipase A2 (56) and provides the free fatty acid substrate for uncoupling proteins. Uncoupling proteins 2 and 3 have ROS sensitive thiol sensors, which when oxidized by superoxide/peroxide generate proteo-glutathionyl mixed disulfides which activate their anti-ROS action (57). The generation of superoxide by SDH and the oxidation of a thiol redox center in the substrate pocket may represent a third ROS-sensing/ROS-reducing feedback loop, which is modulated by sOMF stimulation. It has been demonstrated that oxidation of Cys90 at the substrate pocket leads to the formation of an SDH-glutathionyl mixed disulfide that inhibits function and arrests ROS generation (22). In **Figure 10C** we present a simple scheme that shows how SDH is inactivated by sOMF, based on SDH thiol sensing, with sOMF treatment causing SDH to generate superoxide/peroxide. Either superoxide or hydrogen peroxide in the mitochondrial matrix oxidizes Cys90 of SDH to the sulfenic acid. The cystenic acid would then react with GSH and cause the formation of the SDH-glutathionyl mixed disulphide, blocking the substrate pocket.

In the context of cancer, and cancer therapy therefore one of the reasons for the differential sensitivity to sOMF of cancer cells over ‘normal’ cells becomes apparent. The redox potential of cancer cells is typically predominantly oxidizing, with NAD^+^/NADH and NADP^+^/NADPH ratios, respectively, 5 and 10 times higher than in normal cells (58), which also means that GSSG/GSH ratios are similarly higher (59).

While the present study was conducted to test the hypothesis that the sOMF stimulation produced by the noninvasive anticancer Oncomagnetic Device works at least in part by perturbing the mitochondrial electron transport in SDH or Complex II, one question that may arise is whether the effects observed by us here could also be produced by existing oscillating magnetic field sources in the enivironment. This is unlikely because sOMF produced by our device are super low frequency non-radiating fields that are orders of magnitude stronger than any source in the general environment. For example, the maximum magnetic field strength 12 inches from a standard microwave oven is ~24 µT and 40 feet from a 345 kV high voltage power line is ~5.6 µT. The maximum magnetic field strength of radiating electromagnetic waves such as radiofrequency waves near broadcasting stations and antennas are even weaker. In contrast, the oncoscillators used in our experiments produce a maximum magnetic field strength of ~80 mT at the distance of the mitochondria or cells in the O_2_ electrode chamber. The environmental sources also do not replicate the frequency and timing patterns that are required to produce the mitochondrial effects that we observe.

GBM is currently being treated with some success using Novocure’s Tumor Treating Fields (TTF) device (60). During therapy, electrical fields of 200 kHz frequency are passed through a patient’s head *via* electrodes placed on the scalp. TTF are believed to affect protein dipole-dipole interactions, arresting microtubule spindle formation, disrupting chromosomal mobility during metaphase, leading to mitotic catastrophe, and then to apoptosis of dividing cells (61). sOMF stimulation does not operate in this manner given the cytotoxic effect of sOMF on non-dividing GBM cell cultures subject to 24 h of serum starvation.

To conclude, in this study we provide evidence that specific on and off patterns of sOMF cycles generated by rapid rotation of a strong permanent magnet perturb mitochondrial electron transport as measured by sOMF-induced changes in the time course of O_2_ consumption. This mechanism is likely responsible for sOMF-induced cytotoxicity in DIPG, GBM and meningioma tumor cells. Ongoing experiments are investigating further the utility of this mechanism in producing anticancer effects in mouse xenograft models and end-stage recurrent GBM patients under an FDA-approved expanded access program (18).

## Data Availability Statement

The original contributions presented in the study are included in the article/supplementary material. Further inquiries can be directed to the corresponding authors.

## Ethics Statement

The animal study was reviewed and approved by Houston Methodist Research Institute Institutional Animal Care and Use Committee.

## Author Contributions

MS, SH, OI, DB, and KP designed the study and interpreted its findings. MS and SH drafted the manuscript, which was reviewed and finalized by DB. SH designed the sOMF apparatuses used in the study and supervised their construction and testing. DB provided the patient tumor cells which were harvested during surgery, under an IRB approved protocol, and Peak Brain Tumor Center director. All authors discussed the study results and their implications. All authors contributed to the article and approved the submitted version.

## Funding

Funding for this work was from a Translational Research Initiative grant of the Houston Methodist Research Institute to SH and DB, and from Kenneth R. Peak Foundation, John S. Dunn Foundation, Taub Foundation, Blanche Green Fund of the Pauline Sterne Wolff Memorial Foundation, Kelly Kicking Cancer Foundation, The Methodist Hospital Foundation, Veralan Foundation and many donations in honor of Will McKone. The John S. Dunn Foundation also supports the Distinguished Professorship of MS.

## Conflict of Interest

SH, DB, MS, and KP are listed as inventors on a U.S. patent application filed by Houston Methodist Hospital relating to technology described in this manuscript.

The remaining author declares that the research was conducted in the absence of any commercial or financial relationships that could be construed as a potential conflict of interest.

## Publisher’s Note

All claims expressed in this article are solely those of the authors and do not necessarily represent those of their affiliated organizations, or those of the publisher, the editors and the reviewers. Any product that may be evaluated in this article, or claim that may be made by its manufacturer, is not guaranteed or endorsed by the publisher.

## References

[1] Jimenez H Blackman C Lesser G Debinski W Chan M Sharma S. Use of Non-Ionizing Electromagnetic Fields for the Treatment of Cancer. Front Biosci (Landmark Ed) (2018) 23:284–97. doi: 10.2741/4591 28930547

[2] Helekar SA Hambarde S Ijare OB Pichumani K Baskin DS Sharpe MA. Selective Induction of Rapid Cytotoxic Effect in Glioblastoma Cells by Oscillating Magnetic Fields. J Cancer Res Clin Oncol (2021). doi: 10.1007/s00432-021-03787-0 PMC11801958 34477946

[3] Woodward JR Foster TJ Jones AR Salaoru AT Scrutton NS. Time-Resolved Studies of Radical Pairs. Biochem Soc Trans (2009) 37(Pt 2):358–62. doi: 10.1042/BST0370358 19290862

[4] Woodward JR. Radical Pairs in Solution. Prog React Kinet Mech (2002) 27:165–207. doi: 10.3184/007967402103165388

[5] Eveson RW Timmel CR Brocklehurst B Hore PJ McLauchlan KA. The Effects of Weak Magnetic Fields on Radical Recombination Reactions in Micelles. Int J Radiat Biol (2000) 76(11):1509–22. doi: 10.1080/09553000050176270 11098854

[6] Montoya RD. Magnetic Fields, Radicals and Cellular Activity. Electromagn Biol Med (2017) 36(1):102–13. doi: 10.1080/15368378.2016.1194291 27399314

[7] Werner HJ Schulten K Weller A. Electron Transfer and Spin Exchange Contributing to the Magnetic Field Dependence of the Primary Photochemical Reaction of Bacterial Photosynthesis. Biochim Biophys Acta (1978) 502(2):255–68. doi: 10.1016/0005-2728(78)90047-6 306834

[8] Dismukes GC McGuire A Blankenship R Sauer K. Electron Spin Polarization in Photosynthesis and the Mechanism of Electron Transfer in Photosystem I. Experimental Observations. Biophys J (1978) 21(3):239–56. doi: 10.1016/S0006-3495(78)85522-2 PMC1473678 204369

[9] Closs G. A Mechanism Explaining Nuclear Spin Polarizations in Radical Combination Reactions. J Am Chem Soc (1969) 91:4552–4. doi: 10.1021/ja01044a043

[10] Kaptein R Oosterhoff L. Chemically Induced Dynamic Nuclear Polarization III (Anomalous Multiplets of Radical Coupling and Disproportionation Products). Chem Phys Lett (1969) 4:214–6. doi: 10.1016/0009-2614(69)80105-3

[11] Gilbert BC Davies MJ McLauchlan KA. Electron Paramagnetic Resonance. Cambridge: The Royal Society of Chemistry (2000).

[12] Bittl R Weber S. Transient Radical Pairs Studied by Time-Resolved EPR. Biochim Biophys Acta (2005) 1707(1):117–26. doi: 10.1016/j.bbabio.2004.03.012 15721610

[13] Kaptein R Dijkstra K Nicolay K. Laser Photo-CIDNP as a Surface Probe for Proteins in Solution. Nature (1978) 274(5668):293–4. doi: 10.1038/274293a0 683312

[14] Hore PJ Mouritsen H. The Radical-Pair Mechanism of Magnetoreception. Annu Rev Biophys (2016) 45:299–344. doi: 10.1146/annurev-biophys-032116-094545 27216936

[15] Maffei ME. Magnetic Field Effects on Plant Growth, Development, and Evolution. Front Plant Sci (2014) 5:445. doi: 10.3389/fpls.2014.00445 25237317 PMC4154392

[16] Foley LE Emery P. Drosophila Cryptochrome: Variations in Blue. J Biol Rhythms (2020) 35(1):16–27. doi: 10.1177/0748730419878290 31599203 PMC7328257

[17] Reppert SM Gegear RJ Merlin C. Navigational Mechanisms of Migrating Monarch Butterflies. Trends Neurosci (2010) 33(9):399–406. doi: 10.1016/j.tins.2010.04.004 20627420 PMC2929297

[18] Baskin DS Sharpe MA Nguyen L Helekar SA. Case Report: End-Stage Recurrent Glioblastoma Treated With a New Noninvasive Non-Contact Oncomagnetic Device. Front Oncol (2021) 11:708017. doi: 10.3389/fonc.2021.708017 34367992 PMC8341943

[19] Brand MD Murphy MP. Control of Electron Flux Through the Respiratory Chain in Mitochondria and Cells. Biol Rev Camb Philos Soc (1987) 62(2):141–93. doi: 10.1111/j.1469-185X.1987.tb01265.x 3300795

[20] Chappell JB Hansford RG. 4 - Preparation of Mitochondria from Animal Tissues and Yeasts. In: Birnie GD, editor. Subcellular Components, 2nd ed. London: Butterworth-Heinemann (1972). p. 77–91.

[21] Gutman M Kearney EB Singer TP. Control of Succinate Dehydrogenase in Mitochondria. Biochemistry (1971) 10(25):4763–70. doi: 10.1021/bi00801a025 5140191

[22] Chen YR Chen CL Pfeiffer DR Zweier JL. Mitochondrial Complex II in the Post-Ischemic Heart: Oxidative Injury and the Role of Protein S-Glutathionylation. J Biol Chem (2007) 282(45):32640–54. doi: 10.1074/jbc.M702294200 17848555

[23] Quinlan CL Orr AL Perevoshchikova IV Treberg JR Ackrell BA Brand MD. Mitochondrial Complex II can Generate Reactive Oxygen Species at High Rates in Both the Forward and Reverse Reactions. J Biol Chem (2012) 287(32):27255–64. doi: 10.1074/jbc.M112.374629 PMC3411067 22689576

[24] Ijare OB Baskin DS Sharpe MA Pichumani K. Metabolism of Fructose in B-Cells: A (13)C NMR Spectroscopy Based Stable Isotope Tracer Study. Anal Biochem (2018) 552:110–7. doi: 10.1016/j.ab.2018.04.003 29654744

[25] Sharpe MA Ijare OB Baskin DS Baskin AM Baskin BN Pichumani K. The Leloir Cycle in Glioblastoma: Galactose Scavenging and Metabolic Remodeling. Cancers (2021) 13(8):1815. doi: 10.3390/cancers13081815 33920278 PMC8069026

[26] Orii Y Miki T. Oxidation Process of Bovine Heart Ubiquinol-Cytochrome C Reductase as Studied by Stopped-Flow Rapid-Scan Spectrophotometry and Simulations Based on the Mechanistic Q Cycle Model. J Biol Chem (1997) 272(28):17594–604. doi: 10.1074/jbc.272.28.17594 9211907

[27] Demin OV Kholodenko BN Skulachev VP. A Model of O2.-Generation in the Complex III of the Electron Transport Chain. Mol Cell Biochem (1998) 184(1-2):21–33. doi: 10.1023/A:1006849920918 9746310

[28] Brown GC Brand MD. Thermodynamic Control of Electron Flux Through Mitochondrial Cytochrome Bc1 Complex. Biochem J (1985) 225(2):399–405. doi: 10.1042/bj2250399 2983670 PMC1144603

[29] Gnaiger E Mendez G Hand SC. High Phosphorylation Efficiency and Depression of Uncoupled Respiration in Mitochondria Under Hypoxia. Proc Natl Acad Sci USA (2000) 97(20):11080–5. doi: 10.1073/pnas.97.20.11080 PMC27151 11005877

[30] Hadrava Vanova K Kraus M Neuzil J Rohlena J. Mitochondrial Complex II and Reactive Oxygen Species in Disease and Therapy. Redox Rep (2020) 25(1):26–32. doi: 10.1080/13510002.2020.1752002 32290794 PMC7178880

[31] Korshunov SS Skulachev VP Starkov AA. High Protonic Potential Actuates a Mechanism of Production of Reactive Oxygen Species in Mitochondria. FEBS Lett (1997) 416(1):15–8. doi: 10.1016/S0014-5793(97)01159-9 9369223

[32] Lambert AJ Brand MD. Inhibitors of the Quinone-Binding Site Allow Rapid Superoxide Production From Mitochondrial NADH:Ubiquinone Oxidoreductase (Complex I). J Biol Chem (2004) 279(38):39414–20. doi: 10.1074/jbc.M406576200 15262965

[33] Quinlan CL Perevoshchikova IV Hey-Mogensen M Orr AL Brand MD. Sites of Reactive Oxygen Species Generation by Mitochondria Oxidizing Different Substrates. Redox Biol (2013) 1(1):304–12. doi: 10.1016/j.redox.2013.04.005 PMC3757699 24024165

[34] Murphy Michael P. How Mitochondria Produce Reactive Oxygen Species. Biochem J (2008) 417(1):1. doi: 10.1042/BJ20081386 PMC2605959 19061483

[35] St-Pierre J Buckingham JA Roebuck SJ Brand MD. Topology of Superoxide Production From Different Sites in the Mitochondrial Electron Transport Chain. J Biol Chem (2002) 277(47):44784–90. doi: 10.1074/jbc.M207217200 12237311

[36] Chance B Williams GR. Respiratory Enzymes in Oxidative Phosphorylation. I. Kinetics of Oxygen Utilization. J Biol Chem (1955) 217(1):383–93. 13271402

[37] Chance B Williams GR. The Respiratory Chain and Oxidative Phosphorylation. Adv Enzymol Relat Subj Biochem (1956) 17:65–134. doi: 10.1002/9780470122624.ch2 13313307

[38] Korzeniewski B. 'Idealized' State 4 and State 3 in Mitochondria vs. Rest and Work in Skeletal Muscle. PloS One (2015) 10(2):e0117145–e0117145. doi: 10.1371/journal.pone.0117145 25647747 PMC4412265

[39] Halestrap AP Richardson AP. The Mitochondrial Permeability Transition: A Current Perspective on Its Identity and Role in Ischaemia/Reperfusion Injury. J Mol Cell Cardiol (2015) 78:129–41. doi: 10.1016/j.yjmcc.2014.08.018 25179911

[40] Skulachev VP. Why Are Mitochondria Involved in Apoptosis? Permeability Transition Pores and Apoptosis as Selective Mechanisms to Eliminate Superoxide-Producing Mitochondria and Cell. FEBS Lett (1996) 397(1):7–10. doi: 10.1016/0014-5793(96)00989-1 8941703

[41] Henderson PJ Lardy HA. Bongkrekic Acid. An Inhibitor of the Adenine Nucleotide Translocase of Mitochondria. J Biol Chem (1970) 245(6):1319–26. 4245638

[42] Halestrap AP Brenner C. The Adenine Nucleotide Translocase: A Central Component of the Mitochondrial Permeability Transition Pore and Key Player in Cell Death. Curr Med Chem (2003) 10(16):1507–25. doi: 10.2174/0929867033457278 12871123

[43] Pyrzynska B Serrano M Martínez AC Kaminska B. Tumor Suppressor P53 Mediates Apoptotic Cell Death Triggered by Cyclosporin A. J Biol Chem (2002) 277(16):14102–8. doi: 10.1074/jbc.M104443200 11827957

[44] Qin X Chen Z. Metabolic Dependence of Cyclosporine A on Cell Proliferation of Human Non−Small Cell Lung Cancer A549 Cells and Its Implication in Post−Transplant Malignancy. Oncol Rep (2019) 41(5):2997–3004. doi: 10.3892/or.2019.7076 30896878

[45] Yu W Zhang X Liu J Wang X Li S Liu R. Cyclosporine A Suppressed Glucose Oxidase Induced P53 Mitochondrial Translocation and Hepatic Cell Apoptosis Through Blocking Mitochondrial Permeability Transition. Int J Biol Sci (2016) 12(2):198–209. doi: 10.7150/ijbs.13716 26884717 PMC4737676

[46] Brand MD Nicholls DG. Assessing Mitochondrial Dysfunction in Cells. Biochem J (2011) 435(2):297–312. doi: 10.1042/BJ20110162 21726199 PMC3076726

[47] Sueishi Y Hori M Ishikawa M Matsu-Ura K Kamogawa E Honda Y. Scavenging Rate Constants of Hydrophilic Antioxidants Against Multiple Reactive Oxygen Species. J Clin Biochem Nutr (2014) 54(2):67–74. doi: 10.3164/jcbn.13-53 24688213 PMC3947969

[48] Felberg NT Hollocher TC. Inactivation of Succinate Dehydrogenase by N-Ethylmaleimide. Stoichiometry and Chemistry. J Biol Chem (1972) 247(14):4539–42. 5043853

[49] Lin GL Wilson KM Ceribelli M Stanton BZ Woo PJ Kreimer S. Therapeutic Strategies for Diffuse Midline Glioma From High-Throughput Combination Drug Screening. Sci Trans Med (2019) 11(519):eaaw0064. doi: 10.1126/scitranslmed.aaw0064 PMC7132630 31748226

[50] Mouritsen H Ritz T. Magnetoreception and Its Use in Bird Navigation. Curr Opin Neurobiol (2005) 15(4):406–14. doi: 10.1016/j.conb.2005.06.003 16006116

[51] Huang LS Shen JT Wang AC Berry EA. Crystallographic Studies of the Binding of Ligands to the Dicarboxylate Site of Complex II, and the Identity of the Ligand in the "Oxaloacetate-Inhibited" State. Biochim Biophys Acta (2006) 1757(9-10):1073–83. doi: 10.1016/j.bbabio.2006.06.015 PMC1586218 16935256

[52] Anderson RF Shinde SS Hille R Rothery RA Weiner JH Rajagukguk S. Electron-Transfer Pathways in the Heme and Quinone-Binding Domain of Complex II (Succinate Dehydrogenase). Biochemistry (2014) 53(10):1637–46. doi: 10.1021/bi401630m PMC3985935 24559074

[53] Bulteau AL Ikeda-Saito M Szweda LI. Redox-Dependent Modulation of Aconitase Activity in Intact Mitochondria. Biochemistry (2003) 42(50):14846–55. doi: 10.1021/bi0353979 14674759

[54] Armstrong JS Whiteman M Yang H Jones DP. The Redox Regulation of Intermediary Metabolism by a Superoxide-Aconitase Rheostat. Bioessays (2004) 26(8):894–900. doi: 10.1002/bies.20071 15273991

[55] Lushchak OV Piroddi M Galli F Lushchak VI. Aconitase Post-Translational Modification as a Key in Linkage Between Krebs Cycle, Iron Homeostasis, Redox Signaling, and Metabolism of Reactive Oxygen Species. Redox Rep (2014) 19(1):8–15. doi: 10.1179/1351000213Y.0000000073 24266943 PMC6837700

[56] Madesh M Balasubramanian KA. Activation of Liver Mitochondrial Phospholipase A2 by Superoxide. Arch Biochem Biophys (1997) 346(2):187–92. doi: 10.1006/abbi.1997.0288 9343365

[57] Mailloux RJ Xuan JY Beauchamp B Jui L Lou M Harper ME. Glutaredoxin-2 Is Required to Control Proton Leak Through Uncoupling Protein-3. J Biol Chem (2013) 288(12):8365–79. doi: 10.1074/jbc.M112.442905 PMC3605654 23335511

[58] Moreira JDV Hamraz M Abolhassani M Bigan E Pérès S Paulevé L. The Redox Status of Cancer Cells Supports Mechanisms Behind the Warburg Effect. Metabolites (2016) 6(4):33. doi: 10.3390/metabo6040033 PMC5192439 27706102

[59] Bhowmick R Sarkar RR. Differential Suitability of Reactive Oxygen Species and the Role of Glutathione in Regulating Paradoxical Behavior in Gliomas: A Mathematical Perspective. PloS One (2020) 15(6):e0235204. doi: 10.1371/journal.pone.0235204 32584884 PMC7316271

[60] Mittal S Klinger NV Michelhaugh SK Barger GR Pannullo SC Juhasz C. Alternating Electric Tumor Treating Fields for Treatment of Glioblastoma: Rationale, Preclinical, and Clinical Studies. J Neurosurg (2018) 128:414–21. doi: 10.3171/2016.9.JNS16452 PMC6836465 28298023

[61] Giladi M Schneiderman RS Voloshin T Porat Y Munster M Blat R. Mitotic Spindle Disruption by Alternating Electric Fields Leads to Improper Chromosome Segregation and Mitotic Catastrophe in Cancer Cells. Sci Rep (2015) 5:18046. doi: 10.1038/srep18046 26658786 PMC4676010

